# Nurses’ perspectives on inpatient falls in a large academic hospital in South Africa

**DOI:** 10.4102/curationis.v46i1.2479

**Published:** 2023-10-20

**Authors:** Christine Rogers, Athene Irving

**Affiliations:** 1 Department of Health and Rehabilitation Sciences, Faculty of Health Sciences, University of Cape Town, Cape Town, South Africa

**Keywords:** hospital-based falls, falls risk identification, falls prevention, nursing practice, low- and middle-income country

## Abstract

**Background:**

Falls risk assessment tools, including the Morse Falls Scale, have been used for years, and yet falls remain key adverse events in hospitals. Nurses are key role players in falls prevention and can champion patient safety.

**Objectives:**

The aim of the study was to explore ward nurses’ attitudes, knowledge and practices regarding the use of falls risk assessment tools, institutional falls policy and falls prevention.

**Methods:**

A survey design was used. All permanent ward nurses were eligible to participate, and a convenience sample was used.

**Results:**

Nurses endorsed the Morse Falls Scale, recommended by institutional policy, as effective in reducing falls and indicated that incident reporting measured progress on monitoring fall events. Falls prevention training was scanty; however, nurses were keen for further education of falls.

**Conclusion:**

Effective falls risk management needs to extend beyond promulgating policy and actively address nursing and patient education.

**Contribution:**

This study adds to the sparse literature regarding nursing practice and falls prevention in a developing country. Recommendations for change have been made.

## Introduction

Falls are a global phenomenon and a major contributor to morbidity and mortality (Montero-Odasso et al. 2022). While falls can occur in any setting, falls within a hospital context are serious and increasing, especially in the older adult population (King et al. 2018). Falls are one of the most commonly reported adverse events which occur in hospitals (Haines & Hill 2020). The World Health Organization (WHO) estimates that 80% of falls occur in hospitals and healthcare facilities located in emerging regions (WHO 2021). High-income countries provide evidence for the prevalence and consequences of falls. For example, in the United States alone, between 700 000 and 1 million falls occur in hospitals every year; and overall, 2% of patients will fall during their hospital stay (LeLaurin & Shorr 2019). At least one in four, and possibly up to half the patients who experience a fall in hospital, will sustain injuries (LeLaurin & Shorr 2019). Besides personal injury, falls within a hospital setting incur prolonged hospital stays and expenses, including litigation, so insurers such as Medicare in the United States will not cover costs incurred by an inpatient who falls (LeLaurin & Shorr 2019). Such economic measures should have promoted a sharp reduction in patients’ falls which has not occurred (King et al. 2018).

The term ‘never event’ describes high-cost, high-volume events which should be reasonably prevented with judicious use of evidence-based guidelines (King et al. 2018). Screening and risk prediction therefore make intuitive sense to promote effective prevention. However, the use of formal falls risk assessment tools (FRATs) is controversial, with organisations like the National Institute of Health and Care Excellence (NICE) not recommending the routine use of FRAT in hospitalised patients. Rather, NICE suggests considering all older adults (> 65 years of age) to be at risk for falls and mandates clinicians to be concerned regarding younger patients’ (50–64 years of age) risk of falls as ‘dictated by their condition’ (Swift & Iliffe 2014). The NICE guidelines are being updated and should be released in mid-2024. The World Falls Guidelines mandates all hospitals to have policies, procedures and protocols consistent with best practice in place (Montero-Odasso et al. 2022).

In keeping with the notion of ‘never events’, we would argue that *all* hospitalised patients are vulnerable, frequently immobile, may have reduced cognitive and physical reserve, and therefore are at elevated risk for falls (Fridman 2019). Indeed, the risk of falls continues in discharged patients with many falling within 1 month of leaving the hospital. For example, the risk of hip fracture increases by 100% – 150% in the first month after discharge (Wright et al. 2022). Nurses are ideally placed to make a valuable contribution to lowering the prevalence of hospital-based falls. Nurses’ continuous ward presence and monitoring of both the patient and their physical environment makes them key members of a multidisciplinary team dedicated to reducing fall risk and occurrence. Indeed, in some high-income countries, falls in wards are regarded as a nursing-sensitive quality indicator (Bernet et al. 2022).

Given the high prevalence of falls in industrialised countries’ hospitals, it is a matter of concern that there is scant research reporting the prevalence of falls in low- and middle-income countries (LMICs). Here, nurses are likely less well-resourced and working under difficult conditions. There is a global shortage of nurses, experienced most acutely in LMIC. Unfavourable nursing staff levels are associated with poorer patient safety outcomes, including falls (Imam et al. 2022; Sloane et al. 2018). Nurses from LMICs are likely to have large numbers of patients to care for at any given time. In addition, as nurses are vital in surveilling and reporting adverse events (Imam et al. 2022) including falls, staff shortages might arguably impact the accuracy and even availability of prevalence data. Even though up to 80% of falls occur when patients are not directly observed (Heng et al. 2020), it is clear that the nurse is a vital team member to promote educating patients to take care at all times and call for assistance when mobilising. Falls management must be a team effort in the ward with all role players, including the patient and their loved ones, actively educated, aware and involved.

Given both the constraints of healthcare systems in LMIC combined with the ageing population, it is highly likely that falls in hospitals are occurring and will follow an increasing trajectory as experienced in high-income countries. Nurses are pivotal to champion the prevention of falls, but little is known about their knowledge, attitudes and practices regarding falls in ward settings in LMIC. Therefore, the aim of this study, embedded in a larger study (Irving 2020) concerned with reviewing records of falls and their sequelae experienced by inpatients at a large tertiary hospital in South Africa, was to explore the knowledge, attitudes and practices of ward-based nursing staff towards falls and the institutional falls policy.

## Research methods and design

### Study design

A descriptive cross-sectional survey design was selected using a purposely adapted questionnaire (Appendix 1). The advantage of using a survey is that the research can produce a large amount of empirical data in a short time for a low cost. A disadvantage is that the data may lack depth, nor can the data furnish details of causality (Siedlecki 2020). As this project aimed to gain baseline insight into knowledge, attitudes and practices of nurses, the survey study design was considered most practical.

### Sampling method

A convenience sampling method was adopted.

### Study context and eligible participants

The hospital in which the study was set is a large academic hospital in South Africa. There are 975 beds for all medical and surgical disciplines and the hospital is linked to a research-intensive university. All registered professional nurses (RPNs), enrolled nurses (ENs) and enrolled nursing assistants (ENAs) employed in the inpatient wards at the research site were eligible for inclusion (total of 600). Agency staff were excluded as these nurses may have limited knowledge and exposure to the culture of falls reporting, ward falls prevention practices and falls. Similarly, senior nursing management may have had a different experience of the hospital’s falls policy to nurses working on the wards and was therefore excluded.

### Instrumentation and materials

The questionnaire used and modified for this study was originally developed and used as part of the 6-PACK nursing staff survey (Barker et al. 2016). The latter survey was part of the world’s largest randomised control trial examining falls prevention and nurse-led interventions including fall risk tools and fall alert signs in Australian hospital wards (Barker, Treml & Van der Velde 2017). The questionnaire, informed consent and information documents were translated into Afrikaans and isiXhosa so that nurses who were more comfortable sharing information in either of these languages were not disadvantaged. The latter languages are the most spoken in the province where the survey was conducted.

Demographic information on the length of employment at the hospital, nursing qualification and falls training received was collected. A Likert scale was used to explore the nursing staff’s knowledge of the falls definition and policy, use of the Morse Falls Scale (MFS) (mandated in the institution’s falls policy), ward practice in falls prevention and falls intervention strategies. Finally, there were open-ended questions to identify whether there were features of the falls prevention programme that required improvement; whether barriers to implementation of prevention measures exist, and questions related to training needs in falls prevention.

### Procedure

Institutional permission to conduct the study was granted. After permission from the developers, the selected questionnaire as explained under ‘Instrumentation and materials’ (Appendix 1) was modified, and feedback was sourced from a panel of experts (see section Reliability and validity).

Following electronic communication with the head of nursing, the researcher attended a nursing management meeting 3 weeks before the nursing survey, where the study was introduced to the ward managers.

### Pilot survey

Three weeks prior to commencing the full hospital survey, a pilot was undertaken. The aim of the pilot was to identify any logistical errors in the procedure. Two wards were randomly selected, and research personnel met with the unit managers to introduce the study and provided hard copies of the English, Afrikaans and isiXhosa questionnaires, informed consent forms and information documents. All documents were made available to the nurses on each ward for 1 week. Two clearly marked, sealed boxes were provided to each ward for responses. Post boxes were stored in a safe place on the ward (e.g., the staff common room), and staff deposited their completed questionnaires and consent forms into each box. After 1 week, the researcher returned to the wards to collect the boxes. Nursing unit managers (NUMs) were not involved in distributing or administering the surveys to avoid hierarchical coercion and to ensure strict confidentiality and anonymity of the nurses wishing to participate. The NUMs from both pilot wards requested that a further week be given to allow staff more time to complete the questionnaire. Therefore, the boxes were left for a further week on the wards and collected thereafter.

One week after the completion of the pilot, the main study commenced, with two changes made to the study protocol. Firstly, the time that questionnaires were made available was increased from 1 to 2 weeks. Secondly, the researcher and assistant visited each ward after the first week to ensure copies of all documents were available in each language, remind staff on duty about the study and encourage them to complete the questionnaire.

The main survey commenced and was conducted over 6 weeks to obtain as many responses as possible due to shift changes. A total of 600 consent forms and questionnaires were distributed to the inpatient wards at the hospital. Completed survey responses were then entered into a Microsoft Excel spreadsheet and prepared for analysis.

### Reliability and validity testing for the survey questionnaire

The questionnaire used and modified for this study was originally developed and used as part of the 6-PACK nursing staff survey (Barker et al. 2016). The original version includes 43 questions related to beliefs about falls, current falls prevention practice, best practice guidelines, components of the 6-PACK programme, falls reporting practices and safety climate. Questions relating to fall practice were modified, and questions about the safety climate were removed as the latter were not related to the study’s aims. To check the modified questionnaire for contextual and vocabulary equivalence, a panel of three experts with experience in both clinical and senior management areas and with an in-depth knowledge of the hospital’s falls policy was formally appointed. The panel included the head of the Quality Assurance Department, a chief physiotherapist (who worked with both in- and out-patients) and a representative from the nursing management, all employed at the research hospital and who were not participants in the study. It was deemed important that the time taken to complete the questionnaire should be less than 10 minutes to not interfere with nursing duties and break time. Thus, the panel was also asked for feedback on the time taken to complete the questionnaire. The questionnaire was emailed to the panel, which sent their feedback electronically.

Each panellist took less than 10 minutes to read and complete the questionnaire. No panel members reported that any of the questions were ambiguous or pre-empted them to answer in a specific way. Regarding questions not clearly stated, there were four suggestions, which involved minor word changes.

One panel member suggested stressing that the questionnaire would be anonymous to reduce possible risk of repercussions for participants. Question 17 in the original questionnaire implied that the physiotherapy department at the research site requires a doctor to refer a patient for assessment by a physiotherapist. However, all professionals may refer appropriately. In response to the panel’s suggestions, the wording as noted in Table 1 was changed.

**TABLE 1:** Modified nursing survey questions with corresponding suggestions from the panel.

Question number	Original question	Suggested change
4	On what ward do you most frequently work?	On **which** ward do you most frequently work?
8	Incident reporting provides us with a way of measuring how we are going with patient falls.	Incident reporting provides us with a way of measuring how we are **progressing** with patient falls.
		Incident reporting provides us with a way of measuring how we are **doing** with patient falls.
17	I feel confident to request the doctor’s order for a physiotherapy consult for high-risk patients.	**I feel confident to refer high-risk patients** for a physiotherapy **assessment**.

*Source:* Irving, A., 2020, ‘Factors contributing to falls in a tertiary acute care setting in Cape Town, South Africa: A descriptive study’, master thesis, Faculty of Health Sciences, Division of Physiotherapy, University of Cape Town, Cape Town

Following the finalisation of the questionnaire, the instrument was translated into two local languages. Two healthcare professionals with these languages as their first language translated the English questionnaire, informed consent form and information sheet into isiXhosa and Afrikaans. The isiXhosa and Afrikaans versions were then back-translated into English by a second person unfamiliar with the original document. The researcher then compared the original survey and the back translation to ensure conceptual and vocabulary equivalence (Ozolins et al. 2020). There were no discrepancies with the translation of the Afrikaans survey. There were minor discrepancies in the isiXhosa back translation. Therefore, a meeting was held with both isiXhosa translators where consensus on each item was reached. After that, the isiXhosa version was finalised.

### Data management and analysis

Hard copies were retained by the researcher and stored in a locked cupboard, and the university’s data management policy was adhered to in full. Data from the pilot wards were included in the results due to no significant change in the instrument or processes. Neither nursing staff nor wards were identified by name. The sample was too small to support adequately powered tests and therefore no inferential statistics were performed. Descriptive statistics such as frequency and percentage were used to describe the characteristics of participants.

### Ethical considerations

Ethical approval was obtained from the University of Cape Town Faculty of Health Sciences Human Research Ethics Committee (ref: 874/2016).

The study was designed in accordance with the Declaration of Helsinki (World Medical Association 2013), and the research team actively endorsed the Singapore Statement of Research Integrity (Resnik & Shamoo 2011). All research personnel were required to sign a confidentiality agreement. The risk-benefit ratio was favourable. All participants provided their informed consent, and their privacy and anonymity were respected. To facilitate frank comments without fear of disclosure, participants remained anonymous, and surveys were self-administered. All data were reported as aggregated data to safeguard against the threat of victimisation. Wards were identified under the broad unit terms ‘Medical, Surgical, Psychiatry, Trauma/Emergency Care, Gynaecology and Intensive Care’ and not by individual names to avoid stigmatising of wards.

## Results

### Nursing survey response rate

A total of 137 of the 600 surveys were returned, with a response rate of 22.8%. All 137 were included in the data analysis. However, several surveys were incomplete; therefore, for the nursing staff characteristics section of the survey, *n* ranges from 124 to 133.

### Participants

Table 2 depicts the demographic characteristics of the participants. The majority 91% (121) of nurses had worked at the institution for longer than 1 year, while 61% (82) reported working more than 5 years. Under 10% (12) had worked at the hospital for less than a year. Furthermore, 77.6% (104) of respondents reported working in the same ward for more than a year, yet 70% (93) had not received falls prevention training. Half (65) were RPN. Most returned questionnaires (75%) were from nurses working frequently in the medical (46) and surgical wards (47).

**TABLE 2:** Demographic characteristics of nurses.

Demographic characteristics	*n*	%
**Time employed at this hospital**	133	-
< 4 months	1	0.8
4–12 months	11	8.3
1–5 years	39	29.3
> 5 years	82	61.7
**How long have you worked in this ward**	134	-
< 4 months	12	9.0
4–12 months	18	13.4
1–5 years	49	36.6
> 5 years	55	41.0
**Qualification**	130	-
Registered professional nurse	65	50.0
Staff nurse/enrolled nurse	49	37.7
Enrolled nurse assistant	16	12.3
**Frequently worked ward**	124	-
Surgery	47	37.9
Medicine	46	37.1
ICU	9	7.3
Psychiatry	9	7.3
Trauma	6	4.8
Radiotherapy	4	3.2
Obstetrics and gynaecology	3	2.4
**Received falls prevention training**	133	-
Yes	40	30.0
No	93	70.0
**If received training, how long ago**	37	-
< 6 months	11	29.7
6–12 months	9	24.3
1–2 years	5	13.5
> 2 years	12	32.4

*Source:* Irving, A., 2020, ‘Factors contributing to falls in a tertiary acute care setting in Cape Town, South Africa: A descriptive study’, master thesis, Faculty of Health Sciences, Division of Physiotherapy, University of Cape Town, Cape Town

ICU, intensive care unit.

The responses to the survey questions were divided into five broad categories, which will be presented separately, namely:

- nurses’ experiences of the falls prevention programme
- ward practice of falls prevention
- institutional falls policy
- post-falls procedure
- falls definition.

### Nurses’ experiences of the fall prevention programme

Over half the respondents (59%) believed that the institutional falls policy (viz., MFS on admission, repeated in the event of a fall and completion of an adverse incident report) effectively reduced falls. Supporting this, 80% disagreed with a negatively worded statement that falls risk assessment is a waste of time. Most of the surveyed nurses (82%) believed that incident reporting provides a way of measuring progress on falls prevention. Most participants (83%) reported that the MFS was a useful way to identify at-risk patients, and most (82%) were confident to use the MFS.

### Ward practice of falls prevention measures

**FIGURE 1:**
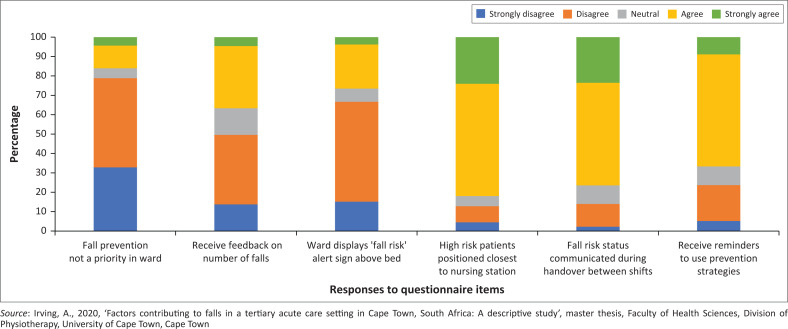
Nurses’ perceptions of ward practice of falls prevention measures.

While only 37% of respondents reported receiving regular feedback on the number of falls occurring in their wards, most (79%) disagreed with a negatively worded statement indicating that falls prevention is a priority on their ward. Less than one-third (27%) of nurses reported using ‘high risk’ signs at the bedside to identify at-risk patients, but most (82%) reported positioning high-risk patients close to the nursing station. Nurses reported that falls risk status is communicated during handover between shifts (77%), and 67% received reminders to use falls prevention strategies (Figure 1).

### Institutional falls policy

Most nurses (86%) believed it was their responsibility to activate the standard care plan for at-risk patients, and similar numbers (89%) agreed that it was their responsibility to update patients’ fall risk status. Almost three-quarters (73%) knew how to complete an adverse incident form required to report falls, and 83% disagreed that they should report falls only when an injury occurs.

### Post-falls procedure

Most nurses (78%) were aware of the post-falls procedure to be followed, and 70% felt confident to refer a patient for physiotherapy.

### Falls definition

**FIGURE 2:**
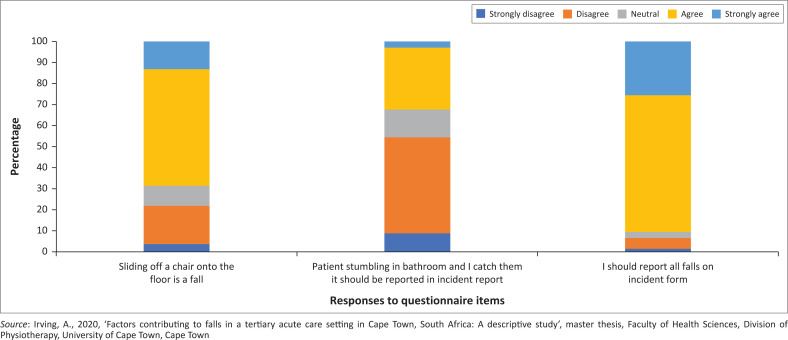
Respondents’ knowledge of the falls definition.

Regarding understanding the definition of a fall, 68% of respondents agreed that sliding off a chair is considered a fall, just less than a third did not agree or were neutral. Only 32% believed that a stumble in the bathroom where the patient is caught should be reported in an incident report, yet most (91%) agreed that all falls should be reported (Figure 2).

### Survey responses to open-ended questions

Responses were grouped into categories to enable analysis. The first question asked nurses’ opinions of what features of the current falls prevention programme required improvement. Just over half the participants responded (*n* = 73, 52.9%). Table 3 identifies that the most reported areas for improvement are staff training in falls prevention (26%), repair and provision of equipment (23.3%) and a review of how falls risk is assessed (15.1%).

**TABLE 3:** Features of the current falls prevention programme that need improvement.

What features of the falls prevention programme need improvement?	*n* (73)	%
Staff training on falls prevention	19	26.0
Repairing and provision of equipment	17	23.3
Review of how falls risk is assessed	11	15.1
All aspects	6	8.2
No features need improvements	6	8.2
There is no falls prevention programme	4	5.7
The use of interventions – there are no *high-risk* signs available	3	4.1
Communication between members of the multidisciplinary team	3	4.1
Feedback to nursing staff regarding fall events	1	1.4

*Source:* Irving, A., 2020, ‘Factors contributing to falls in a tertiary acute care setting in Cape Town, South Africa: A descriptive study’, master thesis, Faculty of Health Sciences, Division of Physiotherapy, University of Cape Town, Cape Town

Challenges reported by the nursing staff included a lack of or faulty equipment (reported by 37% of the respondents), inadequate staff levels and a perceived lack of training in the programme itself. Many staff (66.4%, 91) reported wanting more training and exposure to falls information. Almost half of those who responded (49.4%) requested the training to be in the form of ward-based training, and 37.3% desired workshops. The remaining 12% (11) asked for a combination of written material, workshops and ward training with internet learning.

## Discussion

The discussion will focus on key areas that arose from the survey of nursing staff at the institution. Nurses’ perceptions of training received and desired and environmental factors are discussed before concluding with the strengths and limitations of the study.

### Clinical and training implications

Within a hospital setting, a fall is defined as an ‘unplanned descent to the floor with or without injury to the patient’. The definition specifies that assisted or unassisted falls, in which a staff member attempts to reduce the impact of the fall on the patient (‘catches’), are still regarded as falls (Baernholdt et al. 2017). At the research hospital, the fall definition is congruent with Lamb and colleagues’ seminal definition (Lamb et al. 2005): ‘an event which results in the patient or any part of the patient’s body coming to rest inadvertently on the floor or other surface lower than the patient’. The survey results are interesting because knowledge of what constituted a fall was somewhat patchy. Patients slipping from a chair was recognised as a fall by 68% of the respondents; however, when staff ‘caught’ a potential fall, only 32% of the participants recognised that a fall had occurred. Naturally, this discrepancy would affect reporting of adverse events and might give a distorted picture of patient safety in the wards. Moreover, a shared understanding between patients and the medical and nursing teams as to what constitutes a fall is essential for efforts to reduce the risk of falls to succeed.

Knowledge of risk identification and education has been highlighted as a critical barrier to implementing falls clinical practice guidelines (Montero-Odasso et al. 2022; Mordiffi et al. 2016), and the results of this study would suggest deficits. While 91% (*n* = 121) of nurses had worked at the institution for longer than 1 year, most (70%, *n* = 93) reported they had not received falls prevention training. The fact that at least two of every three nurses in the wards have not had formal training on falls risk identification, and more importantly, falls prevention, is of concern. Thus, implementation of the falls programme must be supported by education of nursing staff and not simply address drawing up of policy. Training for new employees and yearly refresher courses, as regular updates and communication, are important in sustaining practice. The WHO reinforced the importance of educating and skilling healthcare professionals regarding patient safety in order to ‘make zero avoidable harm to patients a state of mind’ (WHO 2021).

Furthermore, an opportunity to promote patient safety exists, as most nurses surveyed (66.4%, *n* = 91) were receptive to falls prevention training. Ward-based training (49.5%) and workshops (37.4%) were the preferred formats for training. Previous research has shown ward-based and experiential falls training to be more effective than training provided in the classroom environment (Mordiffi et al. 2016; Shaw et al. 2020). Evidence suggests that patient and staff education significantly reduces fall occurrence (Morris et al. 2022); however, patients’ involvement is necessary for success (Haines & Hill 2020) and will require significant empowerment both of patients themselves and nurses to feel comfortable in providing patient education.

Turning now to championing falls information in the wards, the results suggest nurses did not perceive that feedback on falls was provided, with only 36.7% reporting feedback is given at the ward level. Lack of responsiveness may have a detrimental impact on nurses’ efforts to improve patient safety. However, it should be noted that in a multilingual and multicultural society such as South Africa, the quality of communication and updates at nursing handover are equally important for patient care (Hada, Coyer & Jack 2018). Moreover, the nuances of power dynamics and challenges facing the nursing profession have to be acknowledged (Hart 2015; Labrague 2021) and are likely exacerbated given the crisis in South African public healthcare and history of apartheid (Malakoane et al. 2020; Malange 2023; Rispel & Bruce 2014). Worryingly, recent literature has linked toxic nursing management styles with increased rates of adverse patient outcomes, including falls (Labrague 2021). Conversely, good communication has repeatedly been found to enhance patient safety (Alanazi, Sim & Lapkin 2022; Fuchshuber & Greif 2022).

### Risk assessment and management

One of the most clinically relevant and surprising findings was that most (83%) nurses surveyed believed the MFS was useful in identifying patients at risk for falls. Such an opinion is contrary to the body of evidence and recommendations. For example, when used in China, an extremely large-scale study found the MFS was limited in its ability to identify hospitalised adults at risk of falls and not cost-effective (Huang et al. 2021), although controversy regarding the MFS persists, as discussed next. Literature reports MFS data for sensitivity ranging from 31% to 98% and specificity between 8% and 97% (Bóriková et al. 2017). Furthermore, studies including a systematic review found the MFS inferior to other measures like STRATIFY in terms of sensitivity (Aranda-Gallardo et al. 2013, De Oliveira Silva et al. 2023). Factors contributing to the wide range of predictive values included the type of ward, length of stay, cut-off scores used and frequency of assessment (Aranda-Gallardo et al. 2013; Bóriková et al. 2017). The settings for most of the studies included in Borikova et al.’s review were high-income countries, likely very different to an LMIC context. However, newer literature from Turkey found the MFS inferior to an alternative measure, upholding the concerns regarding sensitivity and specificity (Kuş, Büyükyilmaz & ARDıÇ 2023) and poor diagnostic performance (De Oliveira Silva et al. 2023). Morse Falls Scale may be capable of identifying patients at high risk for falls, but given that falls are ‘never events’ and may result in litigation (Lee, Seo & Kim 2023), potentially against nurses, one should question if such a psychometrically and diagnostically unstable measure is fit for purpose and is justifiable for use in an institutional falls policy. Indeed, a recent evidence review in a nursing journal (Schoberer et al. 2022) considering older adults in hospitals and long-term care facilities did not recommend FRAT, rather individualised assessment and risk management.

Moreover, patients’ risk for falls varies continuously throughout their hospital stay depending on their medical status and the environment, so clinical judgement must be equivalent and possibly preferable to any FRAT. However, should FRAT be assessed only on admission, and again after a fall, it is possible that staff may fail to realise that fluctuations in the patients’ course may elevate falls risk. Interestingly, a cluster randomised trial regarding the use of clinical reasoning was shown to be non-inferior to formalised fall risk assessment scales (Morris et al. 2021).

Any measures and interventions to assess falls risk should be trialled in situ, even down to the specific type of ward or service and condition of the patients, relevant in LMIC where ageing and frailty are increasing (Ara et al. 2022), rather than adopted from other contexts. Rather than clinging to only conducting FRAT, continued consciousness and management of falls at nursing, institutional and population policy level is required (Schoberer et al. 2022). Finally, unless individualised risk assessment is linked to safety and fall prevention efforts, including patient and staff education, the mere identification of risk is unlikely to impact falls.

### Environmental considerations

The physical environment has been identified as one of the root causes of falls in hospitals (Bernhardt et al. 2022; Swift & Iliffe, 2014). Although not the focus of this study, nurses perceived that environmental factors contribute to patient falls. The lack of and faulty equipment was highlighted as an area of the falls programme that needs improvement (23.3%) and as the most common barrier to the implementation of the falls programme (37.3%). The recent pandemic raised alarm regarding the lamentable condition of many state hospitals in South Africa (Moyo et al. 2021). While the research site was in a functional province and well-funded, one can only speculate regarding the situation in hospitals in provinces with poor track records of fiscal and service delivery issues.

A range of alarm systems and alert devices are available and used internationally. Monitoring systems include motion sensors, video surveillance and pressure sensors, which come at a financial cost at outlay and in terms of ongoing maintenance and training (Oh-Park et al. 2021). Again, there is a lack of convincing evidence for such systems to reduce fall occurrences (Morris et al. 2022). Therefore, such systems should be tested for suitability before purchase. One simple step at the research site would be ensuring the current call bell system is functional and within the patient’s reach when in a chair, in bed or in the bathroom. Mobility aids should be available and always be within patients’ reach. Should electronic health records become a reality, these have been used by machine learning with good effect to trigger alarms to staff when a patient is at risk, or altered risk, for falls. Interestingly, at one site, such a system reduced falls by 39% and with it, attendant costs (Oh-Park et al. 2021).

### Strengths and limitations of the study

The response rate was low, with 22% of all eligible nurses who worked on wards having responded. Agency staff were excluded as there was uncertainty if they were aware of the institutional falls policy, which may impact the results and generalisability of the study. The response rate was disappointing, especially considering the effort made by the researcher to attend meetings and introduce the study to unit managers. The respondents may not be representative of the views of all nurses at the hospital and thus threatens the internal and external validity of the survey results. Volunteer bias may affect the generalisability of results. Respondents may have had a particular interest in fall prevention. Likewise, non-response bias would likely affect the results of this study. The views of those who did not respond are underrepresented in the results. The Hawthorne effect may affect the reliability of the survey results. As respondents were aware their responses were studied, they may have changed their responses to produce more socially desirable results based on their perceptions of research expectations (Berkhout et al. 2022). As the survey was part of a larger study to contextualise a record review of falls within the hospital, open-ended questions were limited. The authors acknowledge that other qualitative methods, such as focus groups, would provide richer information regarding nurses’ attitudes and practices to fall prevention.

A strength of the study was using a previously validated questionnaire with adjustments made during an adaptation and translation process. Thus, it is likely that the questionnaire remained reliable and valid. The opportunity for staff to comment was a valuable addition and would likely have highlighted any pressing issues not captured in the survey. The rigorous translation process ensured that nurses could respond to the survey in their home language, encouraging participation from all nursing staff.

### Recommendations

Firstly, a task team of multiple professional disciplines, including nurses, could be appointed to review and inform institutional, provincial and national policies, guided by the evidence base. If FRATs are to be abandoned, policy must consider context and that nursing staff are frequently overstretched, so falls management strategy has to be quick, simple and of course effective. Secondly, there is an appetite for staff training which should be noted. New staff as well as agency staff should be inducted and advised of the falls policy. Annual refresher courses should be held and attendance highly encouraged. In the wards, regular feedback regarding falls events (including near misses), similar to mortality and morbidity meetings, could explore what could have been done better. Such meetings also offer educational opportunities. While observing the need for falls to become ‘never’ events, a supportive and collaborative effort would enhance the reporting and management of falls and shift the attitude from reporting to preventing. The appointment of falls champions, even across clusters of wards, for example, medical or surgical, could lead such efforts in a non-hierarchical way.

Finally, in an ageing hospital such as the one that hosted the study, it is recommended that environmental reviews be conducted regularly and combined with health and safety reviews to reduce the physical risk of falls.

## Conclusion

This article has described nursing staff’s attitudes, knowledge and practices regarding falls in a tertiary academic hospital in a developing country. The definition of a fall was not universally agreed upon, despite a policy being in place. The MFS was thought to be useful despite evidence to the contrary. On a positive note, falls education was desired and would be welcomed. Falls education of patients and staff alike has been proven to be more effective than many other, more expensive interventions. For an emerging country, educating cadres of staff who champion and continue to educate their colleagues regarding falls risk assessment and prevention is intuitively appealing to build a knowledge base, address and empower patients to be concerned with their safety, and is economically and practically viable.

## APPENDIX 1:

Survey Questionnaire, English Version.

 Thank you for participating in this survey.

 We recognise the importance of your privacy so please note:

- All information collected in this survey **will be anonymous.**
- **Your employer will not know whether you have participated in the survey.**
- No personal details are required, so **we have no way of linking your survey responses to you.**
- All results will be grouped, for example: 70% of nurses strongly agreed that falls prevention is primarily the role of the physiotherapist.

**Please answer the following 6 questions by ticking the appropriate box:**

1. How long have you worked at this hospital?
  □ < 4 months   □ 4–12 months   □ 1–5 years   □ > 5 years
2. How long have you worked on this ward?
  □ < 4 months   □ 4–12 months   □ 1–5 years   □ > 5 years
3. What is your qualification?
  □ Professional nurse   □ Enrolled nurse/Staff Nurse   □ Enrolled nurse assistant___________
4. On which ward do you most frequently work?
  ________________
5. Have you received falls prevention training at this hospital before?
  □ Yes   □ No
6. If you have received falls prevention training at this hospital before, how long ago was it?
  □ < 6 months   □ 6–12 months ago   □ 1–2 years ago   □ > 2 years ago

Please circle the response that **best matches your perceptions/experiences** of the falls prevention and management programme **on the ward where you most frequently work.**

1. The current falls prevention programme is effective at reducing falls on my ward.

**Table:**

A	B	C	D	E
Strongly disagree	Disagree	Neutral	Agree	Strongly agree

2. The Morse Falls Risk Assessment Tool used on my ward is a useful way to identify patients at risk of falling.

**Table:**

A	B	C	D	E
Strongly disagree	Disagree	Neutral	Agree	Strongly agree

3. I feel confident to use the Morse Falls Risk Assessment Tool to identify patients at risk of falling.

**Table:**

A	B	C	D	E
Strongly disagree	Disagree	Neutral	Agree	Strongly agree

4. Falls risk assessment is a waste of time.

**Table:**

A	B	C	D	E
Strongly disagree	Disagree	Neutral	Agree	Strongly agree

5. It is my responsibility to activate the Standard Care Plan for patients I identify as having potential fall risk.

**Table:**

A	B	C	D	E
Strongly disagree	Disagree	Neutral	Agree	Strongly agree

6. It is my responsibility to update my patient’s falls risk status if a fall and/or change in condition occurs.

**Table:**

A	B	C	D	E
Strongly disagree	Disagree	Neutral	Agree	Strongly agree

7. Falls prevention is not a priority on my ward.

**Table:**

A	B	C	D	E
Strongly disagree	Disagree	Neutral	Agree	Strongly agree

8. Incident reporting provides us with a way of measuring how we are progressing with patient falls.

**Table:**

A	B	C	D	E
Strongly disagree	Disagree	Neutral	Agree	Strongly agree

9. I know how to complete an Adverse Incident report using the Adverse Incident Management Tool.

**Table:**

A	B	C	D	E
Strongly disagree	Disagree	Neutral	Agree	Strongly agree

10. I should only report falls in which the patient suffers an injury.

**Table:**

A	B	C	D	E
Strongly disagree	Disagree	Neutral	Agree	Strongly agree

11. As a ward we receive regular feedback with regard to the numbers of patient falls on our ward.

**Table:**

A	B	C	D	E
Strongly disagree	Disagree	Neutral	Agree	Strongly agree

12. If a person slides off their chair to the floor, that is considered a fall.

**Table:**

A	B	C	D	E
Strongly disagree	Disagree	Neutral	Agree	Strongly agree

13. If a patient stumbles in the bathroom and I help them keep their balance and ‘catch them’, I should report this in an incident report.

**Table:**

A	B	C	D	E
Strongly disagree	Disagree	Neutral	Agree	Strongly agree

14. I should report all patient falls on an incident form.

**Table:**

A	B	C	D	E
Strongly disagree	Disagree	Neutral	Agree	Strongly agree

15. On our ward we display a ‘falls risk’ alert sign above the bed to communicate to staff which patients are at risk of falling.

**Table:**

A	B	C	D	E
Strongly disagree	Disagree	Neutral	Agree	Strongly agree

16. On our ward we position high falls risk patients closest to the nursing station.

**Table:**

A	B	C	D	E
Strongly disagree	Disagree	Neutral	Agree	Strongly agree

17. I feel confident to refer high-risk patients for a physiotherapy assessment.

**Table:**

A	B	C	D	E
Strongly disagree	Disagree	Neutral	Agree	Strongly agree

18. If a patient falls on my ward, there is a post fall procedure that must be followed to ensure prompt identification of injuries.

**Table:**

A	B	C	D	E
Strongly disagree	Disagree	Neutral	Agree	Strongly agree

19. Each patient’s falls risk status is communicated during handover report between shifts.

**Table:**

A	B	C	D	E
Strongly disagree	Disagree	Neutral	Agree	Strongly agree

20. I receive regular reminders to use falls prevention strategies.

**Table:**

A	B	C	D	E
Strongly disagree	Disagree	Neutral	Agree	Strongly agree

### Comments:

What features of your current fall prevention programme need improvement?

What barriers do you feel may exist to implementing falls prevention programmes on your ward?

Would you like more training on falls prevention?

If yes, what format would you like this training to take? For example, internet course, workshop, written material, on-ward training.
